# Congenital midline sinus of the upper lip: Evaluating the use of ultrasonography

**DOI:** 10.1002/ski2.170

**Published:** 2022-10-01

**Authors:** Taro Akatsuka, Jun Omatsu, Takuya Miyagawa, Issei Omori, Haruka Kawashima, Noboru Oshima, Shinichi Sato

**Affiliations:** ^1^ Department of Dermatology University of Tokyo Graduate School of Medicine Tokyo Japan; ^2^ Shibuya Ekimae Oshima Dermatology Clinic Tokyo Japan

## Abstract

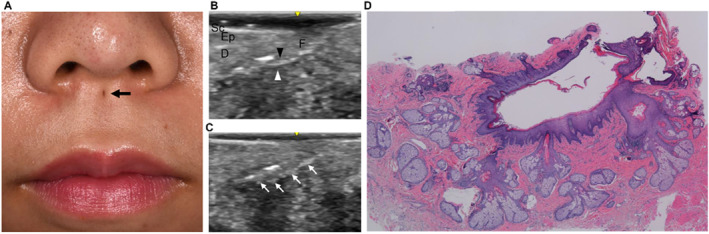

Ultrasound is a minimally invasive examination method. Previous examinations have shown that the most common standard methods used to reveal the distal aspect of the congenital labial sinus, fistula probe or methylene blue dye, are highly invasive and ultrasound is less invasive. Of course, there are cases where CT is necessary, but CT carries the risk of radiation exposure.

## ETHICS STATEMENT

Patients signed the free and informed consent form approved by ethics committee in research, registration number 0695‐(12).

**FIGURE 1:**
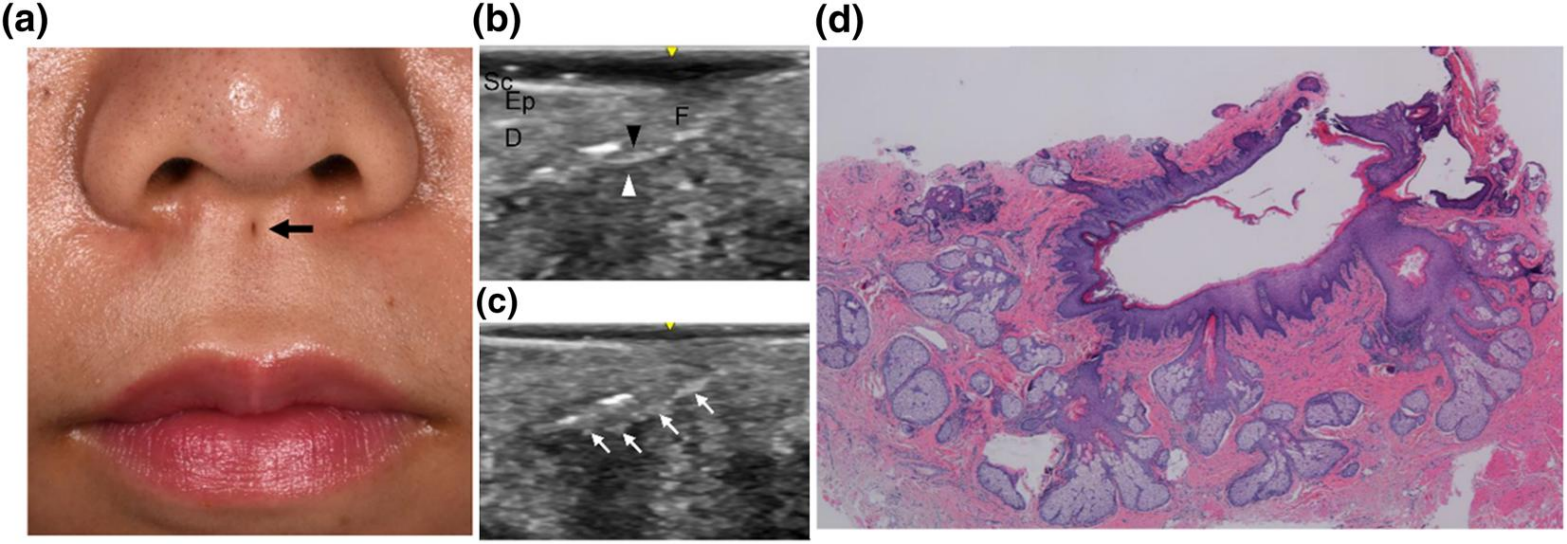
(a) A small midline sinus was noted at the upper lip just below the base of the nasal columella (black arrow). (b), (c) Sonography findings. The tract has two layers; a hyperechoic inner layer (black arrowhead) and a hypoechoic outer layer (white arrowhead). The sebaceous glands are hyperechoic (white arrow). (d) The fistulous tract consisted of cornified stratified squamous epithelium connected with many sebaceous glands (haematoxylin‐eosin, original magnification × 20). Abbreviations: D, dermis; Ep, epidermis; F, fistula; Sc, stratum corneum

Dear Editor,

A congenital lip sinus is a rare malformation occurring in both upper and lower lips, either in isolation or association with congenital deformities, such as a cleft lip and palate in Van der Woude syndrome.^1^ Congential sinus of lower lip has been estimated to be about 0.00001% of the white population. Congential sinus of upper lip are even more uncommom.^2^, ^3^ Despite its rarity, the diagnosis and treatment can be easily made based on its characteristic clinical presentation. To our knowledge, there are about 55 examples,^3^ but there are rare published reports using ultrasonography to assess this disease.^4^ Here, we would like to report the clinical case. A 20‐year‐old woman presented with a dimple in the upper lip philtrum that had been evident since birth (Figure 1a). The upper lip dimple was located at the midline of the philtrum, just below the base of the nasal columella, measuring 2 mm in diameter. A slight colourless, transparent discharge from the upper lip sinus was periodically observed. When gentle pressure was applied, thick creamy sebum‐like discharge could be expressed from the sinus. No congenital abnormalities were noted in the other parts of the body or no family history of orofacial anomalies was reported.

Ultrasonographic examination was performed using a LOGIQ e Premium (GE Healthcare Japan, Tokyo, Japan) equipped with a 22‐MHz linear transducer. A fistulous tract, consisting of a hyperechoic inner layer and hypoechoic outer layer, extended 10 mm towards the anterior nasal spine (Figure 1b). Oval hyperechoic structures were attached to the bottom of the tract (Figure 1c). A diagnosis of a congenital lip sinus was suspected, and complete excision was performed. Histopathology revealed a sinus tract consisting of cornified stratified squamous epithelium connected with many sebaceous glands (Figure 1d), and thus, a diagnosis of a congenital lip sinus was confirmed. No postoperative recurrence was also noted. Prior studies have shown that most cases of midline upper lip were sinus, but some cases were fistulae through the orbicularis.^3^ The most common standard methods used to revealed the distal side, namely fistula probes or methylene blue dye,^2^, ^3^ is highly invasive, while ultrasound is a less invasive testing method.^4^ Of course, CT is sometimes necessary, but CT carries the risk of radiation exposure.^4^ Consistent with the clinical and pathological findings, ultrasonography can be expected to have the following findings; (1) termination of a tract and no connection with the oral or nasal mucosa, (2) a fistula tract composed of a hyperechoic inner layer reflecting the stratum corneum and a hypoechoic outer layer reflecting the epidermis, and (3) hyperechoic or mosaic echoic structures surrounding a tract reflecting the sebaceous glands or other appendages.^5^ In this patient, all these findings were identified by ultrasonographic examination, allowing for preoperative diagnosis and treatment planning. Considering that it is a readily available, noninvasive procedure, ultrasonography can be a very useful tool for the diagnosis and treatment of this rare malformation.

## CONFLICT OF INTEREST

The authors declare that there is no conflict of interest.

## AUTHOR CONTRIBUTIONS

**Taro Akatsuka**: Writing – original draft (Equal). **Jun Omatsu**: Writing – original draft (Equal). **Takuya Miyagawa**: Formal analysis (Equal). **Issei Omori**: Formal analysis (Equal). **Haruka Kawashima**: Formal analysis (Equal). **Noboru Oshima**: Formal analysis (Equal). **Shinichi Sato**: Formal analysis (Equal); Supervision (Lead).

## Data Availability

Data sharing is not applicable to this article as no datasets were generated or analysed during the current study.
